# Long-term efficacy and safety of low-dose rituximab in immune thrombocytopenia: a multicentre, prospective, open-label, randomised controlled trial

**DOI:** 10.1007/s00277-026-06908-2

**Published:** 2026-02-26

**Authors:** Yunfei Chen, Jiaying Liu, Jiawen Dai, Ting Sun, Zijian Qiao, Maosheng Wang, Hu Zhou, Zeping Zhou, Xiaofan Liu, Rongfeng Fu, Feng Xue, Wei Liu, Mankai Ju, Huan Dong, Xinyue Dai, Wenjing Gu, Renchi Yang, Lei Zhang

**Affiliations:** 1 State Key Laboratory of Experimental Hematology, National Clinical Research Center for Blood Diseases, Haihe Laboratory of Cell Ecosystem, Tianjin Key Laboratory of Gene Therapy for Blood Diseases, CAMS Key Laboratory of Gene Therapy for Blood Diseases, Institute of Hematology & Blood Diseases Hospital, Chinese Academy of Medical Sciences & Peking Union Medical College, Tianjin, 300020 China; 2 Tianjin Institutes of Health Science, Tianjin, 301600 China; 3 Langfang Traditional Chinese Medicine Hospital, Langfang, 065099 China; 4 Affiliated Cancer Hospital of Zhengzhou University, Henan Cancer Hospital, Zhengzhou, 450003 China; 5 The Second Affiliated Hospital of Kunming Medical University, Kunming, 650101 China; 6 School of Population Medicine and Public Health, Chinese Academy of Medical Sciences and Peking Union Medical College, Beijing, 100730 China

**Keywords:** Rituximab, Randomised controlled trial, Immune thrombocytopenia, Treatment

## Abstract

Around 60–80% of patients with immune thrombocytopenia (ITP) experience disease relapse after stopping corticosteroids. Rituximab (RTX) is an effective second-line treatment. Low-dose RTX has shown similar efficacy to standard dose, but the optimal dosage remains uncertain. This multicentre, prospective, open-label, randomised controlled trial aimed to compare the long-term efficacy (5-year follow-up) and 1-year safety of two low-dose RTX regimens in Chinese adult patients with glucocorticoid-resistant/dependent or relapsed ITP. Patients (*n* = 104) were randomised 1:1 to group A (RTX 100 mg weekly for 4 weeks) or group B (RTX 375 mg/m^2^ once). The primary outcome was overall response (OR) at 3 months after RTX initiation. Response was followed up until disease relapse or for 5 years. Forty-nine patients in Group A and 51 patients in Group B completed the intervention. At 3 months after RTX initiation, 32 patients in group A (65.3%) and 33 patients in group B (64.7%) achieved OR, and the complete response rate was 42.9% (21/49) in group A and 39.2% (20/51) in group B. The sustained response rates at 6 months, 1, 2, and 5 years were 59.2%, 42.9%, 28.6%, and 20.4% in group A and 58.8%, 43.1%, 33.3%, and 17.6% in group B. These variables were not statistically different between two groups. The most common adverse events were upper respiratory tract and pulmonary infections. RTX 375mg/m^2^ once showed similar long-term efficacy compared to RTX 100 mg weekly for 4 weeks, with a comparable 1-year safety profile; both being well tolerated. The single-dose regimen may be preferable due to greater patient convenience and reduced economic burden.

Trial registration

ClinicalTrials.gov Identifier- NCT01719692 (Registration date: October 26, 2012).

**Supplementary Information:**

The online version contains supplementary material available at 10.1007/s00277-026-06908-2.

## Introduction

 Immune thrombocytopenia (ITP) is an autoimmune hemorrhagic disorder caused by accelerated platelet destruction and impaired platelet production [1]. While most patients respond to glucocorticoids, only 20–40% achieve sustained response after stopping treatments [2–4]. 

Rituximab (RTX) offers an option for those seeking drug-free remission or refusing splenectomy [5]. RTX 375 mg/m^2^ weekly for 4 weeks is considered the standard dosage, with a short-term response of 60–70% [6, 7]. Subsequent studies showed that a reduced dosage of 100 mg/m^2^ weekly for 4 weeks or 375 mg/m^2^ given once achieve similar efficacy [8–10]. However, no studies have focused on the long-term efficacy and safety of these two regimens.

## Methods

### Study design

This multicentre, prospective, open-label, randomised controlled trial (ClinicalTrials.gov ID: NCT01719692) was aimed to compare the long-term efficacy and safety of two low-dose RTX regimens in adult Chinese patients with glucocorticoid-resistant/dependent or relapsed ITP. The patients were enrolled from four hospitals in China. Group A received RTX 100 mg weekly for four weeks, and group B received RTX 375 mg/m^2^ once. The ethics committees of all participating centres approved this study. Informed consent was obtained from each patient in accordance with the principles of the Declaration of Helsinki.

### Patients

The inclusion criteria were 18–60 years of age regardless of sex; diagnosis of primary ITP according to the guidelines of the American Society of Hematology [11] ≥ 3 months in duration; platelet count < 30 × 10^9^/L (measured at least twice during the screening, with at least a 1-week interval); and failed or dependent on or relapsed after previous treatment with glucocorticoid; Additional key inclusion criteria were as follows: if on glucocorticoid maintenance therapy, dose ≤ 0.5 mg/kg prednisone or equivalent and stabilised for ≥ 4 weeks; drugs such as azathioprine, danazol, and cyclosporine A stopped for ≥ 4 weeks; splenectomy ≥ 6 months previously; rescue therapy completed ≥ 2 weeks before administration. Key exclusion criteria included secondary thrombocytopenia, previous treatment with RTX or allergic to RTX, uncontrollable primary diseases of important organs, HIV-positive status, active infection (including hepatitis B, hepatitis C, and other viral antigens or DNA, RNA positive) or bleeding, and thrombotic disease. The criteria for withdrawal and termination of the study included severe adverse event (AE); severe bleeding with platelet count < 10 × 10^9^/L, also ineffective to limited rescue therapy; no response for ≥ 3 months and other treatment required; and death, severe infection, or other life-threatening situation. Full criteria are shown in Supplementary Appendix 1.

### Treatment strategies

SPSS program 20.0 (IBM Corporation, Armonk, NY, USA) was used to generate random numbers. Each number represented one of the two regimens (group A, RTX 100 mg once weekly for 4 weeks; group B, RTX 375 mg/m^2^ once) in a 1:1 ratio. All random numbers were kept in sealed envelopes and distributed to each centre. The investigators of each centre selected the patients to be included in the groups and treated them according to the instructions of the number. Clinicians, investigators, and patients were not blinded to the group assignments. After initiating RTX treatment, if the patient had at least two consecutive evaluations (interval > 7 days) without rescue treatment and a platelet count > 50 × 10^9^/L, the concomitant medications could be reduced or stopped. Within 3 months of the last RTX dose, rescue therapy is recommended if the patient has an extremely low platelet count and an obvious bleeding tendency. Rescue therapy was limited to IVIG and glucocorticoids.

### Follow-up

Routine blood tests were performed weekly within 3 months of RTX initiation, every 2 weeks in 3–6 months, every month in 6–12 months, every 2 months in the second year, and every 3 months starting from the third year. Three months after RTX initiation, the World Health Organization (WHO) bleeding score [12] and reduction in concomitant medications were assessed. Within 1 year of RTX initiation, hepatitis B virus DNA quantification, liver and kidney function tests, electrolytes, electrocardiogram, urinary and stool analyses, and evaluations for AEs were performed monthly according to the Common Terminology Criteria for Adverse Events (CTCAE, version 4.0). For response to RTX treatment, patients were followed up until recurrence or 5 years.

### Study endpoints

Efficacy was evaluated in accordance with the ITP international consensus [13]. Complete response (CR) was defined as a platelet count ≥ 100 × 10^9^/L without bleeding. Partial response (PR) was defined as a platelet count ≥ 30 × 10^9^/L and at least two times higher than the baseline count without bleeding. Overall response (OR) was defined as CR or PR. No response (NR) was defined as a platelet count < 30 × 10^9^/L, an increase in the platelet count to less than two times the baseline value, or bleeding. Relapse was defined as a platelet count < 30 × 10^9^/L after any response, a decrease in the platelet count to less than two times the baseline value, or bleeding symptoms. Sustained response (SR) was defined as patient efficacy maintained for > 6 months after the initial treatment. When defining response, it should be tested at least two times with an interval of ≥ 7 days. When defining relapse, it should be tested at least two times with an interval of ≥ 1 day.

The primary outcome was OR at 3 months after RTX initiation. The secondary endpoints included CR at 3 months after RTX initiation; SR over 6 months and 1, 2, 3, 4, and 5 years after RTX initiation; time to response (TTR), time from RTX initiation to response; relapse-free survival (RFS), the interval between RTX initiation and relapse in patients who achieved OR; received rescue treatment within 3 months after RTX initiation; the change in concomitant medication and the change in WHO bleeding score at 3 months after RTX initiation; and the incidence of AEs.

### Statistical analysis

This study was designed as a prospective, non-inferiority trial. Based on our previous findings [14], we estimated an overall response rate (ORR) of 58% for Group A and 50% for Group B at 3 months. A non-inferiority margin of 20% was chosen, reflecting the maximum clinically acceptable loss of efficacy in exchange for the convenience of a single-dose regimen. Assuming a 5% dropout rate, 52 patients per group were required to achieve 80% power with a one-sided alpha of 0.05 to demonstrate non-inferiority. Categorical variables were compared using Pearson’s chi-square test or Fisher’s exact test. Continuous variables were compared using the Mann-Whitney U test, Kruskal-Wallis H test, or one-way analysis of variance. The Kaplan-Meier method and log-rank test were used to compare relapse-free patients between groups. Statistical significance was set at *P* < 0.05. The Bonferroni method was used to adjust P values for multiple comparisons. Before regression analysis for factors associated with OR at 3 months after RTX initiation, restricted cubic spline analysis was used to determine the cutoff values of baseline variables (age, body mass index, platelet count, and IgG, IgA, and IgM levels). Binary logistic regression was used for univariable analysis, and variables with *P* < 0.2 were included in the multivariate analysis. Multivariable analysis was performed using binary logistic backward stepwise regression. Data were analysed using SPSS 16.0 and R 3.5.0.

The intention-to-treat set included all randomly assigned patients. The full analysis set (FAS) included patients who received RTX at least once and had platelet counts. The per-protocol analysis set included patients who completed the treatment without major protocol violations. The safety set (SS) included patients who received RTX at least once and were assessed for safety. Baseline characteristics and primary and secondary endpoints were analysed in the FAS. Safety was analysed in the SS.

## Results

### Patient characteristics

**Fig. 1:**
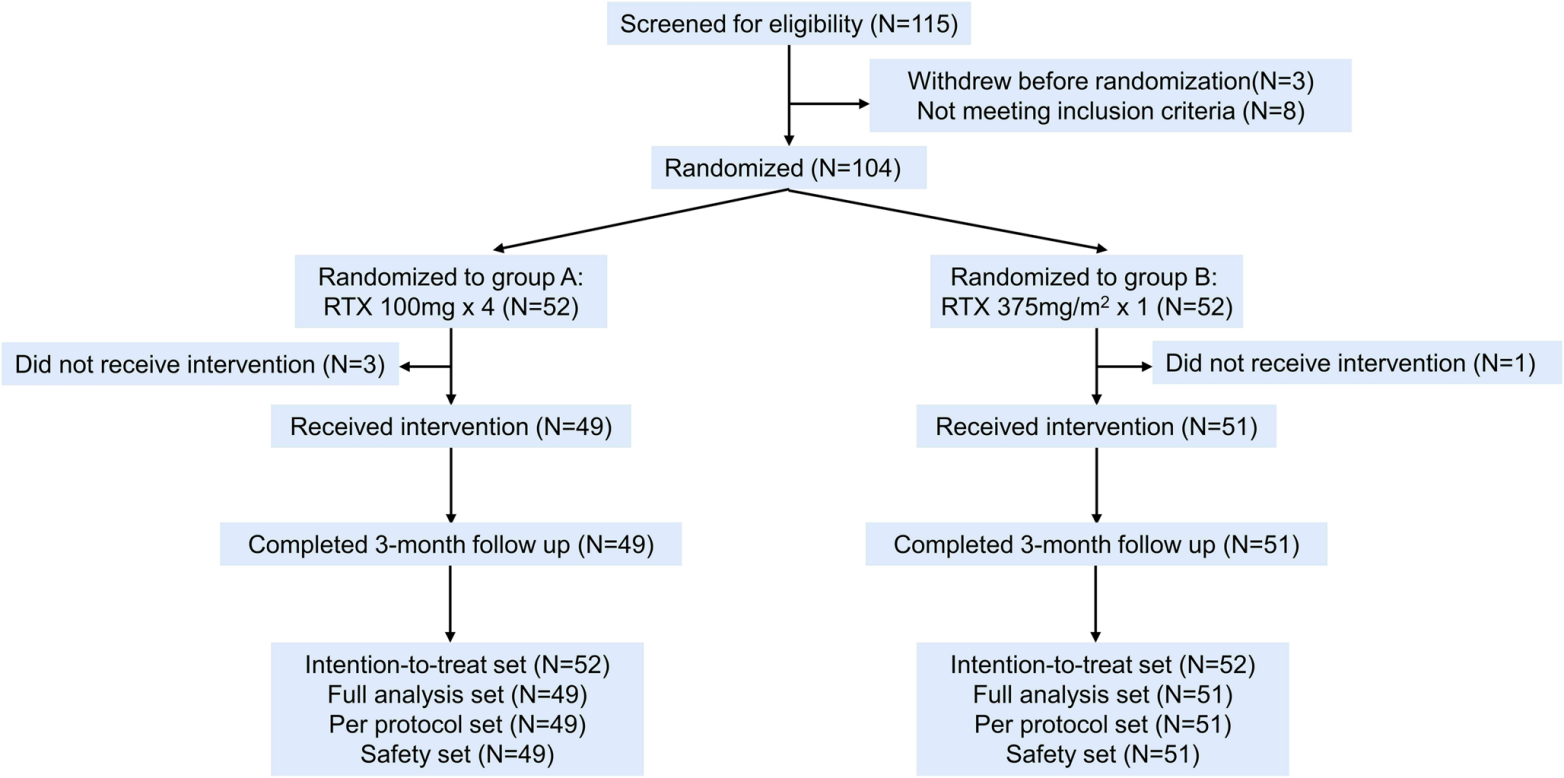
Study design

Patient enrolment began in August 2012 and ended in October 2015. Of the 115 patients screened, 11 were excluded (3 withdrew before randomization, 8 did not meet the inclusion criteria). Fifty-two patients were randomized to group A (100 mg weekly for 4 weeks) and 52 to group B (375 mg/m^2^ once). Three patients in group A and one in group B did not receive treatment. Ultimately, 49 patients in group A and 51 patients in group B received treatment and completed the 3-month follow-up (Fig. 1). Table 1 shows the baseline characteristics in the FAS. The median age of group A and group B was 31 years and 32 years, respectively. Females comprised 69.4% (34/49) of group A and 62.7% (32/51) of group B. The median baseline platelet count was 11 × 10^9^/L in both groups. The median time from ITP diagnosis to medication was 18 months for group A and 27 months for group B. All patients had previously received glucocorticoids. The baseline WHO bleeding score of both groups ranged from 0 to 2, mainly 0–1. There were no statistically significant differences between groups in platelet membrane glycoprotein antibody status, Ig levels, or other baseline variables.

**Table 1:** Baseline characteristics in different RTX groups (the full analysis set)

Baseline characteristics	Group A (100 mg x 4) (*N* = 49)	Group B (375mg/m^2^ × 1) (*N* = 51)	*P*
Age, year, median (IQR)	31.0 (20.5–45.5)	32.0 (23.0–40.0)	0.90
Female, n (%)	34 (69.4)	32 (62.7)	0.48
BMI, kg/m^2^, mean ± SD	23.00 ± 2.72	23.43 ± 2.38	0.41
BSA, m^2^, mean ± SD	1.66 ± 0.18	1.67 ± 0.17	0.62
Platelet count, x10^9^/L, median (IQR)	11.0 (6.0–17.0)	11.0 (6.0–18.0)	0.58
ITP duration, month, median (IQR)	18.0 (12.0–60.0)	27.0 (10.0–72.0)	0.76
Previous therapies, n (%)	/	/	/
Glucocortecoids	49 (100.0)	51 (100.0)	/
IVIG	26 (53.1)	33 (64.7)	0.24
Cyclosporin	7 (14.3)	7 (13.7)	0.94
Vindesine	11 (22.4)	9 (17.6)	0.55
rhIL-11	10 (20.4)	6 (11.8)	0.24
Danazol	11 (22.4)	12 (23.5)	0.90
MMF	1 (2.0)	1 (2.0)	0.98
rhTPO	12 (24.5)	8 (15.7)	0.27
CTX	1 (2.0)	1 (2.0)	0.98
Splenectomy	1 (2.0)	1 (2.0)	0.98
No. of previous treatment, median (IQR)	3 (2–4)	2 (2–3)	0.58
GC use at baseline	41 (83.7)	38 (74.5)	0.26
Bleeding score, n (%)	/	/	0.40
0	22 (44.9)	16 (31.4)	
1^a^	24 (49.0)	30 (58.8)	
2^b^	3 (6.1)	5 (9.8)	
GPAb^c^ status, n (%)	/	/	
GPIIbIIIaAb positive	30 (61.2)	30 (58.8)	0.81
GPIbIXAb positive	14 (28.6)	16 (31.4)	0.76
GPIaIIa positive	11 (22.4)	11 (21.6)	0.92
Serum immune globulin	/	/	/
IgG, g/L, median (IQR)	10.7 (8.9–12.6)	11.7 (8.8–14.7)	0.34
IgA, g/L, median (IQR)	1.8 (1.2–2.2)	1.5 (1.2–2.5)	0.81
IgM, g/L, median (IQR)	1.3 (0.8–1.6)	1.1 (0.7–1.4)	0.13

a. Grade 1: Mild bleeding such as skin bleeding, petechiae, ecchymosis, nosebleed or gingival bleeding, etc

b. Grade 2: severe patches of ecchymosis, symptomatic or bleeding oral vesicles, significantly increased menstruation, epistaxis > 30 minutes, hematuria, hematemesis, black stool or mild anemia caused by bleeding

c. Platelet glycoprotein (GP) autoantibodies were detected using a commercial kit (Pak Autoassay; Immucor GTI Diagnostics, Waukesha, WI 53186, USA)

*IVIG* intravenous Immunoglobin, *rhIL-11* recombinant human interleukin-11, *MMF* mycophenolate mofetil, *rhTPO* recombinant human thrombopoietin, *CTX* Cyclophosphamide, *GPAb* platelet membrane glycoprotein antibody

### Responses

**Fig. 2:**
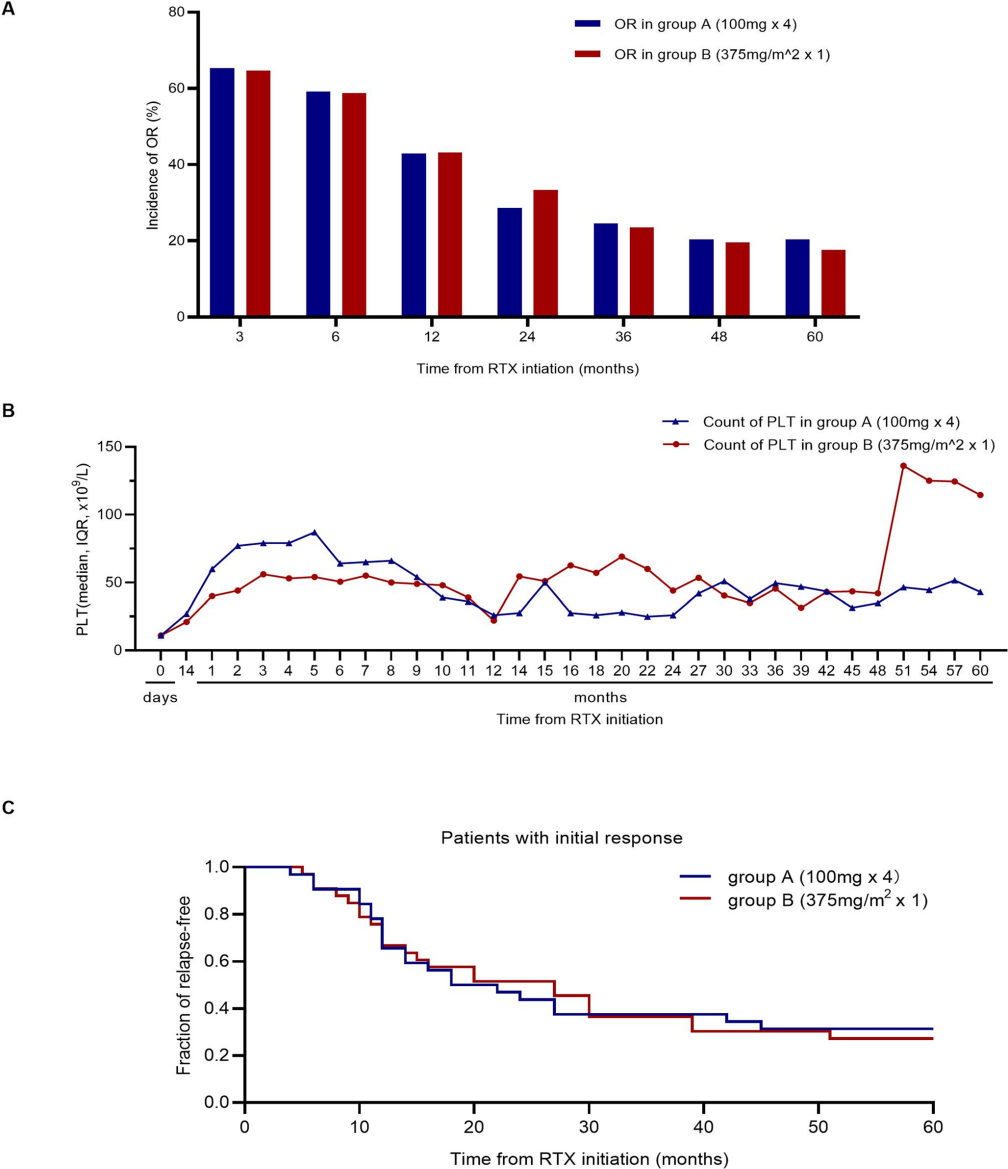
Response in group A and group B. (**A**) Rate of overall response (OR) in the two groups. There was no difference in ORR between two groups at different time points. (**B**) Platelet counts in the two groups. (**C**) Kaplan-Meier plot of time to relapse in patients with an initial response. The Kaplan-Meier curves demonstrated that there was no difference in relapse-free time between the two groups (*P* = 0.89)

Table 2 presents the study endpoints in the FAS. Figure 2A shows the ORR at different follow-up points. For the primary endpoint, OR was achieved in 32 patients in group A (65.3%) and 33 patients in group B (64.7%), without a statistically significant difference (*P* = 0.95). The absolute difference in ORR between the two groups was − 0.6% (95% CI: −18.9% to 19.9%) in the per-protocol population. Consistent results were observed in the intention-to-treat (ITT) population, showing an absolute difference of 1.9% (95% CI: −16.1% to 19.9%), which further supported the non-inferiority of the single-dose regimen. CRR (group A, 21/49 [42.9%]; group B, 20/51 [39.2%]; *P* = 0.71) and PRR (group A, 11/49 [22.4%]; group B, 13/51 [25.5%]; *P* = 0.72) also showed no statistically significant difference. SRR between the two groups was also not statistically significantly different (see Table 2). The median TTR was 3.0 weeks (IQR, 2.0–4.0) for both groups (*P* = 0.61). Improvement in bleeding scores at 3 months after RTX initiation was not statistically significant different (group A, 21/27 [77.8%]; group B, 25/35 [71.4%]; *P* = 0.57). Rescue treatment was needed by 10 patients in group A (20.4%) and 8 patients in group B (15.7%) within 3 months after RTX initiation, with no statistically significant difference (*P* = 0.54). At RTX initiation, 41 patients in group A (83.7%) and 38 patients in group B (74.5%) were on glucocorticoids; 24 (58.5%) and 21 (55.3%) stopped after RTX treatment, respectively, with no statistically significant difference (*P* = 0.77). Platelet counts at each follow-up point are shown in Fig. 2B. Group A’s platelet count increased faster during the first 3 months after RTX initiation, while group B showed a higher platelet count in later follow-ups. This late-stage fluctuation occurred without changes in concomitant therapies and is likely attributed to the increased sensitivity of the median value within a small cohort (*n* = 15). As individual data points in long-term follow-up are subject to clinical variability, such fluctuations are expected when the sample size diminishes.

**Table 2:** Responses and outcomes in different RTX groups (the full analysis set)

	Group A (100 mg x 4) (*N* = 49)	Group B (375mg/m^2^ × 1) (*N* = 51)	*P*
Initial response (3 months)	/	/	/
OR	32 (65.3)	33 (64.7)	0.95
CR	21 (42.9)	20 (39.2)	0.71
PR	11 (22.4)	13 (25.5)	0.72
Sustained response	/	/	/
≥ 6 months	29 (59.2)	30 (58.8)	0.97
≥ 1 year	21 (42.9)	22 (43.1)	0.98
≥ 2 years	14 (28.6)	17 (33.3)	0.61
≥ 3 years	12 (24.5)	12 (23.5)	0.91
≥ 4 years	10 (20.4)	10 (19.6)	0.92
≥ 5 years	10 (20.4)	9 (17.6)	0.73
TTR, weeks, median (IQR)	(*N* = 32) 3.0 (2.0–4.0)	(*N* = 33) 3.0 (2.0–4.0)	0.61
Bleeding score >0 at baseline	27 (55.1)	35 (68.6)	0.16
bleeding score decreased at 3 months	21/27 (77.8)	25/35 (71.4)	0.57
Need rescue therapies	10 (20.4)	8 (15.7)	0.54
GC	1 (2.0)	3 (5.9)	0.64
IVIG	9 (18.4)	8 (15.7)	0.72
Platelet transfusion	4 (8.2)	6 (11.8)	0.49
GC use at baseline	41 (83.7)	38 (74.5)	0.26
GC stopped at 3 months	24/41 (58.5)	21/38 (55.3)	0.77

*OR* overall response, *CR* complete response, *PR* partial response, *TTR* time to response, *GC* Glucocorticoid, *IVIG* intravenous Immunoglobin

RFS was analysed in RTX responders. Figure 2C shows a Kaplan-Meier plot of the time from RTX initiation to relapse. The median RFS was 20.0 months in group A and 27.0 months in group B. The frequency of RFS did not statistically significantly differ between the two groups (*P* = 0.89).

Logistic regression analysis identified factors related to ORR at 3 months after RTX initiation (Table S1). Higher IgA levels (> 1.58 g/L, odds ratio, 3.052; 95% CI, 1.059–8.794; *P* = 0.04) and GPIIbIIIaAb positivity (odds ratio, 7.423; 95% CI, 2.001–27.535; *P* = 0.003) were associated with achieving OR. Prior use of recombinant human thrombopoietin (odds ratio, 0.118; 95% CI, 0.029–0.481; *P* = 0.003), ITP duration ≥ 10 years (odds ratio, 0.170; 95% CI, 0.033–0.886; *P* = 0.04), and GPIbIXAb positivity (odds ratio, 0.108; 95% CI, 0.025–0.462; *P* = 0.003) were linked to failure to achieve OR.

As there is a relationship between GPAb and outcomes, the outcomes of patients with different GPAb statuses were analysed. Table S2 shows that CRR was higher in GPIIbIIIaAb-positive patients (34/60 [56.7%]) compared to GPIIb/IIIaAb-negative patients (7/40 [17.5%]) (*P* < 0.001). The median time to response was also shorter for GPIIbIIIaAb-positive patients (2.0 weeks) than for GPIIbIIIaAb-negative patients (4.0 weeks) (*P* = 0.004). Outcomes with statistically significant differences included SR ≥ 1 year and 4 years, with the GPIIbIIIaAb-positive group performing better. Table S3 shows the outcomes of patients with different GPIbIXAb statuses. PRR at 3 months after RTX initiation was statistically significantly different between GPIbIXAb-negative (22/70 [31.4%]) and GPIbIXAb-positive (2/30 [6.7%]) groups (*P* = 0.008). GPIaIIaAb-positive and GPIaIIaAb-negative patients showed similar outcomes (Table S4).

### Adverse events

Adverse events were systematically monitored and recorded for one year following the initiation of RTX treatment in both groups. The AEs that occurred during follow-up are shown in Table 3. In group A, 27 patients (55.1%) reported AEs, with six (12.2%) experiencing grade ≥ 3 AEs, but no deaths. In group B, 30 patients (58.8%) reported AEs, six (11.8%) had grade ≥ 3 AEs without deaths, and two (3.9%) died. Both deaths occurred in patients refractory to RTX treatment. One died from a pulmonary infection three months after RTX initiation, which was considered potentially related to the combined effect of immunosuppression and refractory disease status. The other died from cerebral hemorrhage one year after starting RTX due to persistent severe thrombocytopenia. These events were adjudicated as not directly caused by drug-specific toxicity but rather reflecting the complications of refractory ITP. Common AEs included upper respiratory tract infections (6/49 [12.2%] in group A and 8/51 [15.7%] in group B) and pulmonary infections (5/49 [10.2%] in group A and 4/51 [7.8%] in group B). Treatment-related AEs occurred in 19/49 (38.8%) in Group A and 19/51 (37.3%) in Group B. There were no statistically significant differences in the incidence of total AEs or specific types of AEs between the two groups.

**Table 3:** Adverse events (the safety set)

AEs	Group A (100 mg x 4) (*N* = 49)	Group B (375mg/m^2^ × 1) (*N* = 51)	*P*
Any AEs	27 (55.1)	30 (58.8)	0.71
Grade ≥ 3	6 (12.2)	6 (11.8)	0.94
Deaths	0	2 (3.9)	0.10
Types	/	/	/
Pulmonary infection	5 (10.2)	4 (7.8) ^a^	0.95
Upper respiratory infection	6 (12.2)	8 (15.7)	0.62
Urinary system infeciton	2 (4.1)	2 (3.9)	> 0.99
Herpes zoster	0	3 (5.9)	0.26
Appendicitis	0	1 (2.0)	0.24
Conjunctivitis	2 (4.1)	0	0.09
Diarrhea	4 (8.2)	3 (5.9)	0.96
Elevated bilirubin	1 (2.0)	1 (2.0)	0.98
Elevated ALT	3 (6.1)	4 (7.8)	> 0.99
Elevated AST	1 (2.0)	4 (7.8)	0.383
Hypopotassemia	3 (6.1)	3 (5.9)	> 0.99
Fatigue	4 (8.2)	4 (7.8)	> 0.99
Headache	3 (6.1)	3 (5.9)	> 0.99
Transfusion reaction ^b^	2 (4.1)	3 (5.9)	> 0.99
Rash	2 (4.1)	1 (2.0)	0.97
Menometrorrhagia	4 (8.2)	5 (9.8)	> 0.99
Gastrointestinal hemorrhage	2 (4.1)	1 (2.0)	0.97
Cerebral hemorrhage	0	1 (2.0) ^c^	0.24
Treatment-related AEs ^d^	19 (38.8)	19 (37.3)	0.88

a. Including one death

b. Transfusion reaction including chest tightness, chills and fever

c. Including one death

d. Treatment-related AEs including pulmonary infection, upper respiratory infection, urinary system infection, herpes zoster, appendicitis, conjunctivitis, diarrhea, transfusion reaction and rash

*AE* adverse effects, *ALT* alanine transaminase, *AST* aspartate aminotransferase

## Discussion

This multicentre, prospective, randomised controlled trial evaluated the long-term efficacy and safety of low-dose RTX regimen (100 mg once weekly for 4 weeks versus 375 mg/m^2^ once) in ITP patients. To ensure a clear evaluation of RTX’s efficacy, no prophylactic treatments that could influence platelet counts, such as IVIG, were permitted in our study. Overall, there were no significant differences in primary and secondary outcomes between the groups. The ORR was approximately 65% and CRR was 40%. Notably, our results showed that the 3-month ORR difference was − 0.6% (95% CI: −18.9% to 19.9%). Since the lower bound of the 95%CI did not exceed the pre-specified non-inferiority margin of 20%, the the non-inferiority of the single-dose regimen to the weekly regimen was statistically confirmed.

Wu et al. [15] reported a 59% ORR and 41% SRR using RTX 100 mg weekly for 6 weeks in glucocorticoid-resistant or relapsed ITP patients, with a follow-up period of one year. Zaja et al. [16] conducted a long-term follow-up study of low-dose RTX (100 mg weekly for 4 weeks) in Italian adult ITP patients, reporting a moderately lower short-term response rate (52% vs. our 65%) and a comparable long-term response rate of 24% (vs. 20% in our study). Ni et al. [17] also compared the efficacy of low-dose (100 mg weekly for 4 weeks, LD-RTX group) and single-dose (375 mg/m^2^, S-RTX group) RTX over a maximum follow-up period of 18 months, finding no significant differences between the two groups. The ORR in our study was consistent with theirs; however, our CRR was higher (42.9% vs. 23.8% in the LD-RTX group, 39.2% vs. 28.3% in the S-RTX group). Owing to the clearance of RTX from the body and the recovery of B cells, some patients with ITP may experience relapse after 6 months of RTX treatment. Our study showed similar SR over 6 months and 1 year compared to Ni’s study, and further demonstrated that there was no statistically significant difference in SR over 5 years between the two regimens. An observational study on the long-term effectiveness of standard-dose RTX demonstrated SR rates of 38%, 31%, and 21% at 1, 2, and 5 years, respectively [18]. These results are consistent with our findings, indicating that low-dose RTX regimens achieve similar long-term efficacy as standard-dose RTX.

Clinical implications of platelet glycoprotein antibody status: One of the most clinically significant findings of this study is the predictive value of GPAb profiling in ITP patients treated with RTX. Our data demonstrate that GPIIbIIIaAb-positive patients had significantly higher CRR and faster response times, while GPIbIXAb-positivity was a predictor of treatment failure. Prior studies have demonstrated that the absence of platelet autoantibodies is associated with ineffective RTX treatment [19]. GPIIbIIIa-positive patients have shown better response to RTX [20], as confirmed by our previous study [21]. RTX can also effectively reduce the level of GPIIbIIIaAb in patients with ITP [22]. A retrospective study indicated that GPIbIXAb-positive patients responded poorly to dexamethasone-RTX treatment [23], which aligns with our findings. Additionally, GPIbIXAb-positive patients have been reported to be unresponsive to IVIG or glucocorticoids, suggesting that these patients have refractory ITP [22]. These findings suggest that GPAb status could serve as a potential biomarker for personalized therapy: GPIIbIIIaAb-positive patients appear to be ideal candidates for RTX, while the poor response in GPIbIXAb-positive patients suggests that alternatives like TPO-RAs or splenectomy should be prioritized. However, the routine clinical application of GPAb profiling faces challenges, primarily due to the lack of standardized, high-sensitivity assays across centers. Therefore, while GPAb status is a powerful biomarker, it must be integrated with other clinical factors, such as disease duration and prior treatment history, to optimize therapeutic decision-making. Future prospective trials are needed to validate these biomarkers within a formal treatment algorithm.

Other factors were also related to the response to RTX. Patients with ITP for over 10 years were less responsive to RTX, suggesting resistant ITP. Patients with IgA levels > 1.58 g/L responded better to RTX than patients with IgA ≤ 1.58 g/L. Previous studies indicated that ITP patients with high IgA levels may be more susceptible to conventional treatments and to splenectomy [24, 25], though the relevant mechanism requires further exploration.

The follow-up for safety was as long as 1 year in our study, with 50% of patients reporting AEs, which was consistent with a previous report [15]. The most common AEs were upper respiratory tract and pulmonary infections, with an incidence of approximately 10%. Most of the infected patients were treated with glucocorticoids when enrolled in the trial. The incidence of infection in low-dose RTX-treated patients for autoimmune haematological diseases has been reported as 30–40% [26]. The overall incidence of infection in our study was similar to that reported in a previous study [15]. In our study, the incidence of infections did not differ significantly between groups, suggesting that the single-dose 375 mg/m² regimen does not result in a more profound or prolonged immunosuppressive state. However, the two deaths in Group B warrant consideration. The death from cerebral hemorrhage was related to refractory disease, while the pulmonary infection at 3 months occurred during the transient B-cell depletion window. Given that our study was not powered to detect rare mortality events, these results should be interpreted with caution.

In the current era where TPO-RAs have become a mainstay of second-line therapy for ITP, the role of RTX requires re-evaluation. Conceptually, RTX and TPO-RAs represent two distinct treatment paradigms. TPO-RAs typically require continuous administration to maintain platelet counts, which can lead to high long-term medical costs and potential concerns regarding long-term safety and patient compliance. In contrast, RTX offers a finite treatment duration, with the potential for inducing long-term drug-free remission. Regarding patient preference, many patients prioritize a ‘one-and-done’ or finite approach. Therefore, our findings support that simplified RTX dosing remains a relevant and cost-effective strategy for patients seeking long-term remission without the need for chronic medication.

Our study had some limitations. First, the open-label design may have introduced potential biases in patient follow-up and the assessment of subjective secondary endpoints, such as bleeding scores. Second, our study was conducted in Chinese adult patients, which may limit the generalizability of the findings to other ethnic or demographic groups. Third, our trial was conducted prior to the widespread adoption of TPO-RAs as second-line therapy, which may be a potential limitation regarding generalizability to current practice. Fourth, although baseline immunoglobulin levels were measured, systematic and longitudinal monitoring was not a mandated part of the long-term follow-up protocol. This represents a limitation, as we were unable to perform an in-depth analysis of the changes in these variables after treatment. Finally, while efficacy was tracked for five years, the safety assessment was capped at one year. While the most common AEs of RTX occur within the first year, long-term risks such as late-onset neutropenia or persistent hypogammaglobulinemia might not be fully captured. Future prospective studies with extended safety monitoring and routine immune profiling are warranted to further refine the safety profile of these simplified RTX regimens.

In summary, low-dose RTX showed good long-term efficacy and safety in patients with glucocorticoid-resistant or -dependent ITP. Administration of a single dose of RTX at 375 mg/m^2^ was equivalent to 100 mg RTX per week for 4 weeks. Given the similar efficacy and safety, the single-dose regimen may be preferable considering patient convenience and economic burden.

## Supplementary Information

Below is the link to the electronic supplementary material.

Supplementary Material 1 (PDF 200 KB)

## Data Availability

All relevant data are within the article and its Supporting Information files.
