# Supplementary material for: Long-term efficacy and safety of low-dose rituximab in immune thrombocytopenia: a multicentre, prospective, open-label, randomised controlled trial
Source: Ann Hematol. 2026 Feb 26;105(4):140. doi: 10.1007/s00277-026-06908-2 (PMC12935823; doi:10.1007/s00277-026-06908-2)
Supplement: Supplementary file 1 — Supplementary Material 1 (PDF 200 KB) [file 277_2026_6908_MOESM1_ESM.pdf]

Supplementary Materials for

Long-term efficacy and safety of low-dose rituximab in immune thrombocytopenia: A multicentre, prospective, open-label, randomised controlled trial

Supplementary Appendix 1. Inclusion, exclusion and withdrawal/termination criteria

Inclusion criteria:

1. Age 18-60 years old;
2. Diagnosis of primary immune thrombocytopenia according to the guidelines of American Society of Hematology for at least 3 months before inclusion;
3. Platelet count <30×10^9/L (measured at least twice during the screening, with at least a 1-week interval);
4. Failed or dependent on or relapsed after previous treatment with glucocorticoid;
5. If on glucocorticoid maintenance therapy, dose ≤0.5 mg/kg prednisone or equivalent and stabilised for at least 4 weeks;
6. Drugs such as azathioprine, danazol, cyclosporine A, tacrolimus, and sirolimus stopped for at least 4 weeks;
7. Splenectomy more than 6 months previously;
8. Previous rescue therapy of ITP (including methylprednisolone, platelet transfusion and IVIG) completed at least 2 weeks before the first administration;
7. Liver and kidney function (including alanine aminotransferase, aspartate aminotransferase, total bilirubin, serum creatine, urea nitrogen, etc.) less than 1.5 times the upper limit of normal value;
8. Eastern Cooperative Oncology Group performance status ≤2;
9. Cardiac function classification (New York Heart Association) ≤2;
10. Understand the research procedure and voluntarily sign a written informed consent form.

Exclusion criteria:

1. Patients with secondary thrombocytopenia (including myelodysplastic syndrome, aplastic anemia, common variant immunodeficiency disease, hereditary thrombocytopenia, drug-induced thrombocytopenia, pseudothrombocytopenia, connective tissue disease secondary thrombocytopenia, thrombocytopenia after liver disease, etc.);
2. Previous treatment of RTX or allergic to RTX;
3. Uncontrollable primary diseases of important organs (including malignant tumor, liver failure, heart failure, kidney failure and other diseases);
4. HIV-positive status;
5. Active infection including hepatitis B (HBV), hepatitis C (HCV) and other viral antigens or DNA, RNA positive; cytomegalovirus, Epstein-Barr virus, syphilis

chronic or active infection. If HBV core antibodies are positive, HBV-DNA testing is required.

	Include	Exclude
HBV	HBsAg negative	HBsAg positive
	HBsAg negative & HBcAb positive & HBV-DNA negative	HBsAg negative & HBcAb positive & HBV-DNA positive
HCV	Antibodies negative	Antibodies positive

6. Extensive and severe bleeding, such as hemoptysis, upper gastrointestinal hemorrhage, intracranial hemorrhage, etc.
7. Heart disease or arrhythmia need treatment, or poorly controlled hypertension;
8. Thrombotic diseases including pulmonary embolism, thrombosis, atherosclerosis, etc.;
9. Previously allogeneic stem cell transplantation or organ transplantation;
10. Mental disorders who are unable to obtain informed consent normally and participate in trials and follow-up;
11. Symptoms of toxicity from pre-trial treatment have not resolved;
12. Other severe conditions that may limit participation in the trial (e.g., diabetes; severe cardiac insufficiency; myocardial infarction or unstable arrhythmia or unstable angina within the last 6 months; gastric ulcer; active autoimmune diseases, etc.);
13. Sepsis or other irregular bleeding;
14. Taking antiplatelet drugs;
15. pregnancy, suspected pregnancy (urine human chorionic gonadotropin positive during screening) or lactation.

Withdrawal/termination criteria

1. Severe AEs or intolerance of treatment which could not be alleviated.;
2. Severe bleeding and platelet count lower than 10×10^9/L, and ineffective limited rescue therapy and need to be treated with other regimens (including splenectomy and other immunosuppressants);
3. No response for more than 3 months, other treatment options are required;
4. Death, severe infection, bleeding or other life-threatening situations;
5. Pregnancy;
6. Violate this trial protocol;
7. Other situations including the researcher's belief that the study needs to be

terminated.

8. patient request to terminate treatment.

Table S1. Logistics regression analysis of factors associated with overall response at 3 months after RTX initiation (the full analysis set)

Factors	Univariable analysis		Multivariable analysis	
	OR (95% CI)	*P* value	OR (95% CI)	*P* value
Female (ref: male)	1.816 (0.771-4.277)	**0.17**	/	/
Age ＞31 years (ref: ≤31 years)	1.462 (0.639-3.346)	0.37	/	/
BMI ＞23.67 kg/m^2 (ref: ≤23.67 kg/m^2)	1.027 (0.451-2.336)	0.95	/	/
Previous glucocortecoid-response (ref: no response)	1.548 (0.388-6.182)	0.53	/	/
Previous glucocortecoid-complete response	2.466 (0.855-7.114)	**0.10**	/	/

(ref: no complete response)				
Previous IVIG<br>(ref: no IVIG)	1.347 (0.586-3.096)	0.48	/	/
Previous CsA<br>(ref: no CsA)	0.678 (0.215-2.140)	0.51	/	/
Previous VDS<br>(ref: no VDS)	1.000 (0.358-2.794)	>0.99	/	/
Previous rhIL-11<br>(ref: no rhIL-11)	0.474 (0.161-1.397)	**0.18**	/	/
Previous danazol<br>(ref: no danazol)	1.012 (0.381-2.691)	0.98	/	/
Previous MMF<br>(ref: no MMF)	NA	>0.99	/	/

Previous rhTPO<br>(ref: no rhTPO)	0.351 (0.129-0.955)	**0.04**	0.118 (0.029-0.481)	**0.003**
Previous CTX<br>(ref: no CTX)	NA	>0.99	/	/
Previous splenectomy<br>(ref: no splenectomy)	0.531 (0.032-8.761)	0.66	/	/
Comorbidities<br>(ref: no comorbidities)	1.091 (0.341-3.487)	0.88	/	/
Previous bleeding<br>(ref: no bleeding)	NA	>0.99	/	/
ITP duration<br>(ref: ＜1 year)	/	/	/	/
1 year ≤ ITP duration ＜ 2 years	1.412 (0.409-4.870)	**0.59**	2.512 (0.584-10.804)	0.22

2 years ≤ ITP duration < 5 years	1.235 (0.353-4.320)	**0.74**	1.215 (0.298-4.955)	0.79
5 year ≤ ITP duration < 10 years	1.376 (0.371-5.102)	**0.63**	1.221 (0.264-5.646)	0.80
ITP duration ≥ 10 years	0.294 (0.076-1.145)	**0.08**	0.170 (0.033-0.886)	**0.04**
RTX group B<br>(ref: A)	0.974 (0.428-2.216)	0.95	/	/
Bleeding at baseline<br>(ref: no bleeding)	0.430 (0.174-1.059)	**0.07**	/	/
Baseline: platelet count > 10 x 10^9/L<br>(ref: ≤10 x 10^9/L)	2.538 (1.089-5.918)	**0.03**	/	/
Baseline: IgG > 11.21 g/L<br>(ref: ≤11.21 g/L)	0.643 (0.281-1.472)	0.30	/	/
Baseline: IgM > 1.1 g/L<br>(ref: ≤1.1 g/L)	1.092 (0.480-2.484)	0.83	/	/

Baseline: IgA ＞1.58 g/L (ref: ≤1.58 g/L)	1.862 (0.808-4.290)	**0.14**	3.052 (1.059-8.794)	**0.04**
GPIIbIIIaAb positive (ref: negative)	1.725 (0.748-3.976)	**0.20**	7.423 (2.001-27.535)	**0.003**
GPIbIXAb positive (ref: negative)	0.490 (0.203-1.182)	**0.11**	0.108 (0.025-0.462)	**0.003**
GPIaIIaAb positive (ref: negative)	1.200 (0.437-3.293)	0.72	/	/

IVIG: intravenous immunoglobin; CsA: cyclosporin; VDS: vindesine; rhIL-11: recombinant human interleukin-11; MMF: mycophenolate mofetil; rhTPO: recombinant human thrombopoietin; CTX: Cyclophosphamide; GPAb: platelet membrane glycoprotein antibody.

Table S2. Responses and outcomes in GPIIbIIIaAb negative/positive patients (the full analysis set)

	GPIIbIIIaAb- (N=40)	GPIIbIIIaAb+ (N=60)	*P*
Initial response (3 months)	/	/	/
OR	23 (57.5)	42 (70.0)	0.20
CR	7 (17.5)	34 (56.7)	**<0.001**
PR	16 (40.0)	8 (13.3)	**0.002**
Sustained response	/	/	/
⩾6 months	19 (47.5)	40 (66.7)	0.06
⩾1 year	10 (25.0)	33 (55.0)	**0.003**
⩾2 years	10 (25.0)	21 (35.0)	0.29
⩾3 years	6 (15.0)	18 (30.0)	0.09
⩾4 years	4 (10.0)	16 (26.7)	**0.04**
⩾5 years	4 (10.0)	15 (25.0)	0.06
TTR, weeks, median (IQR)	(N=23) 4.0 (3.0-4.0)	(N=42) 2.0 (2.0-4.0)	**0.004**
Bleeding score ＞0 at baseline	27 (67.5)	35 (58.3)	0.36
bleeding score decreased at 3 months	20/27 (74.1)	26/35 (74.3)	0.99
Need rescue therapies	7 (17.5)	11 (18.3)	0.92
GC	3 (7.5)	1 (1.7)	0.35
IVIG	7 (17.5)	10 (16.7)	0.91

Platelet transfusion	3 (7.5)	7 (11.7)	0.73
GC use at baseline	30 (75.0)	49 (81.7)	0.42
GC stopped at 3 months	13/30 (43.3)	32/49 (65.3)	0.06

OR: overall response; CR: complete response; PR: partial response; TTR: time to response; GC: Glucocorticoid; IVIG: intravenous Immunoglobin.

Table S3. Responses and outcomes in GPIbIXAb negative/positive patients (the full analysis set)

	GPIbIXAb- (N=70)	GPIbIXAb+ (N=30)	*P*
Initial response (3 months)	/	/	/
OR	49 (70.0)	16 (53.3)	0.11
CR	27 (38.6)	14 (46.7)	0.45
PR	22 (31.4)	2 (6.7)	**0.008**
Sustained response			
⩾6 months	44 (62.9)	15 (50.0)	0.23
⩾1 year	30 (42.9)	13 (43.3)	0.97
⩾2 years	24 (34.3)	7 (23.3)	0.28
⩾3 years	19 (27.1)	5 (16.7)	0.26
⩾4 years	16 (22.9)	4 (13.3)	0.28
⩾5 years	15 (21.4)	4 (13.3)	0.34
TTR, weeks, median (IQR)	(N=49) 3.0 (2.0-4.0)	(N=16) 3.0 (2.0-4.0)	0.78
Bleeding score ＞0 at baseline	40 (57.1)	22 (73.3)	0.13
bleeding score decreased at 3 months	30 (75.0)	16 (72.7)	0.85
Need rescue therapies	12 (17.1)	6 (20.0)	0.73
GC	3 (4.3)	1 (3.3)	>0.99
IVIG	12 (17.1)	5 (16.7)	0.95

Platelet transfusion	5 (7.1)	5 (16.7)	0.28
GC use at baseline	53 (75.7)	26 (86.7)	0.22
GC stopped at 3 months	33 (62.3)	12 (46.2)	0.17

OR: overall response; CR: complete response; PR: partial response; TTR: time to response; GC: Glucocorticoid; IVIG: intravenous Immunoglobin.

Table S4. Responses and outcomes in GPIaIIaAb negative/positive patients (the full analysis set)

	GPIaIIaAb- (N=78)	GPIaIIaAb+ (N=22)	*P*
Initial response (3 months)	/	/	/
OR	50 (64.1)	15 (68.2)	0.72
CR	28 (35.9)	13 (59.1)	0.05
PR	22 (28.2)	2 (9.1)	0.06
Sustained response			
⩾6 months	44 (56.4)	15 (68.2)	0.32
⩾1 year	30 (38.5)	13 (59.1)	0.08
⩾2 years	23 (29.5)	8 (36.4)	0.54
⩾3 years	19 (24.4)	5 (22.7)	0.87
⩾4 years	16 (20.5)	4 (18.2)	>0.99
⩾5 years	15 (19.2)	4 (18.2)	>0.99
TTR, weeks, median (IQR)	(N=50)<br>3.0 (2.0-4.0)	(N=15)<br>3.0 (2.0-4.0)	0.81
Bleeding score > 0 at baseline	50 (64.1)	12 (54.5)	0.41
bleeding score decreased at 3 months	37/50 (74.0)	9/12 (75.0)	>0.99
Need rescue therapies	15 (19.2)	3 (13.6)	0.77
GC	3 (3.8)	1 (4.5)	>0.99
IVIG	15 (19.2)	2 (9.1)	0.43

Platelet transfusion	7 (9.0)	3 (13.6)	0.81
GC use at baseline	62 (79.5)	17 (77.3)	>0.99
GC stopped at 3 months	35/62 (56.5)	10/17 (58.8)	0.86

OR: overall response; CR: complete response; PR: partial response; TTR: time to response; GC: Glucocorticoid; IVIG: intravenous Immunoglobin.
